# Tocilizumab in JIA patients who have inadequate response to anti-tumour necrosis factor therapy

**DOI:** 10.1186/1546-0096-9-S1-P186

**Published:** 2011-09-14

**Authors:** R Russo, M Katsicas

**Affiliations:** 1 Service of Immunology & Rheumatology, Hospital de Pediatría Prof. Dr. Juan P. Garrahan, Buenos Aires, Argentina

## Background

Tocilizumab (TCZ), an IL-6 receptor inhibitor, improves arthritis and systemic symptoms associated with systemic JIA. It may be a valuable option in patients with JIA who show inadequate response to anti-TNF agents.

## Aim

To analyze the short-term effectiveness and safety of TCZ in patients with JIA refractory to anti-TNF agents.

## Methods

All patients who received TCZ due to refractory disease were included. All patients had exhibited anti-TNF primary or secondary failure. Data were retrieved from the Rheumatology Section data base. TCZ was administered intravenously at a dose of 8 mg/kg for patients > 30 Kg, 12 mg/Kg for patients < 30 Kg every 2 weeks. Efficacy endpoints included improvement (ACR Pedi30), disappearance of systemic symptoms, and reduction in corticosteroid dose.

## Results

9 patients with JIA (7 systemic, 2 poliarticular, 7 F, 2 M) were included. Median age at study entry was 10 years, disease duration 6 years. They received TCZ for 3 to 9 months. Patients had been refractory to etanercept (9), adalimumab (7) or infliximab (2). At baseline (medians): active joints: 11, joints with limited motion: 4, wellbeing (0-3): 0.1, disease activity (0-3): 0.45, ESR: 35 mm/h; CHAQ > 0.5 3 patients, fever/rash 1 patient. Six patients were receiving corticosteroids. All patients met improvement criteria, 7 before the 3rd TCZ infusion. At 1 month after TCZ therapy began: active joints: 4, joints with limited motion: 3, wellbeing: 0.05, disease activity: 0.24, ESR: 3 mm/h; CHAQ > 0.5 1 patient, fever/rash 0 patient. During the treatment, 4 patients reduced dose (< 50%) of corticosteroids. No side effects were recorded.

## Conclusions

Tocilizumab seems to be effective and safe with a rapid onset of action in children with anti-TNF refractory JIA.

